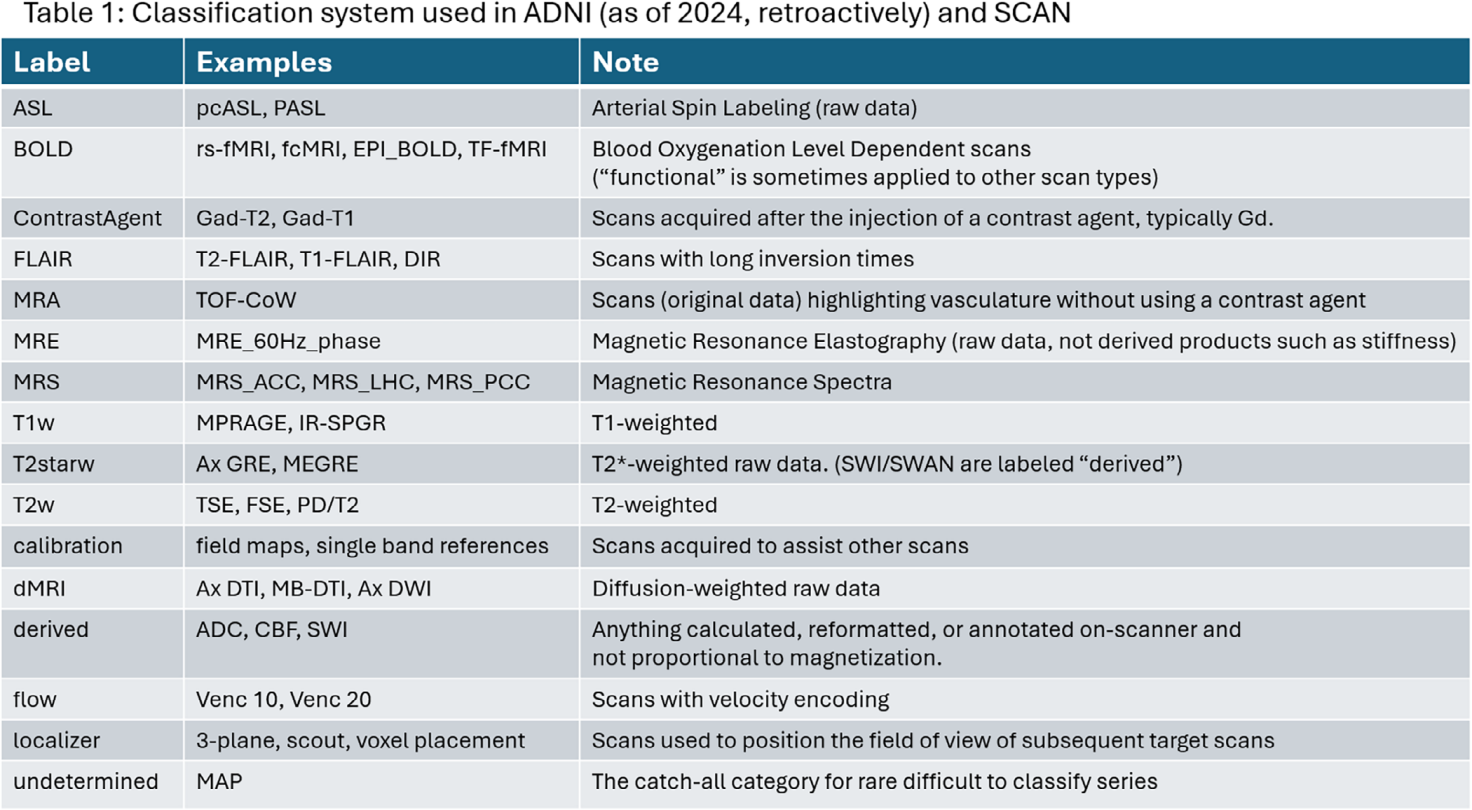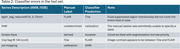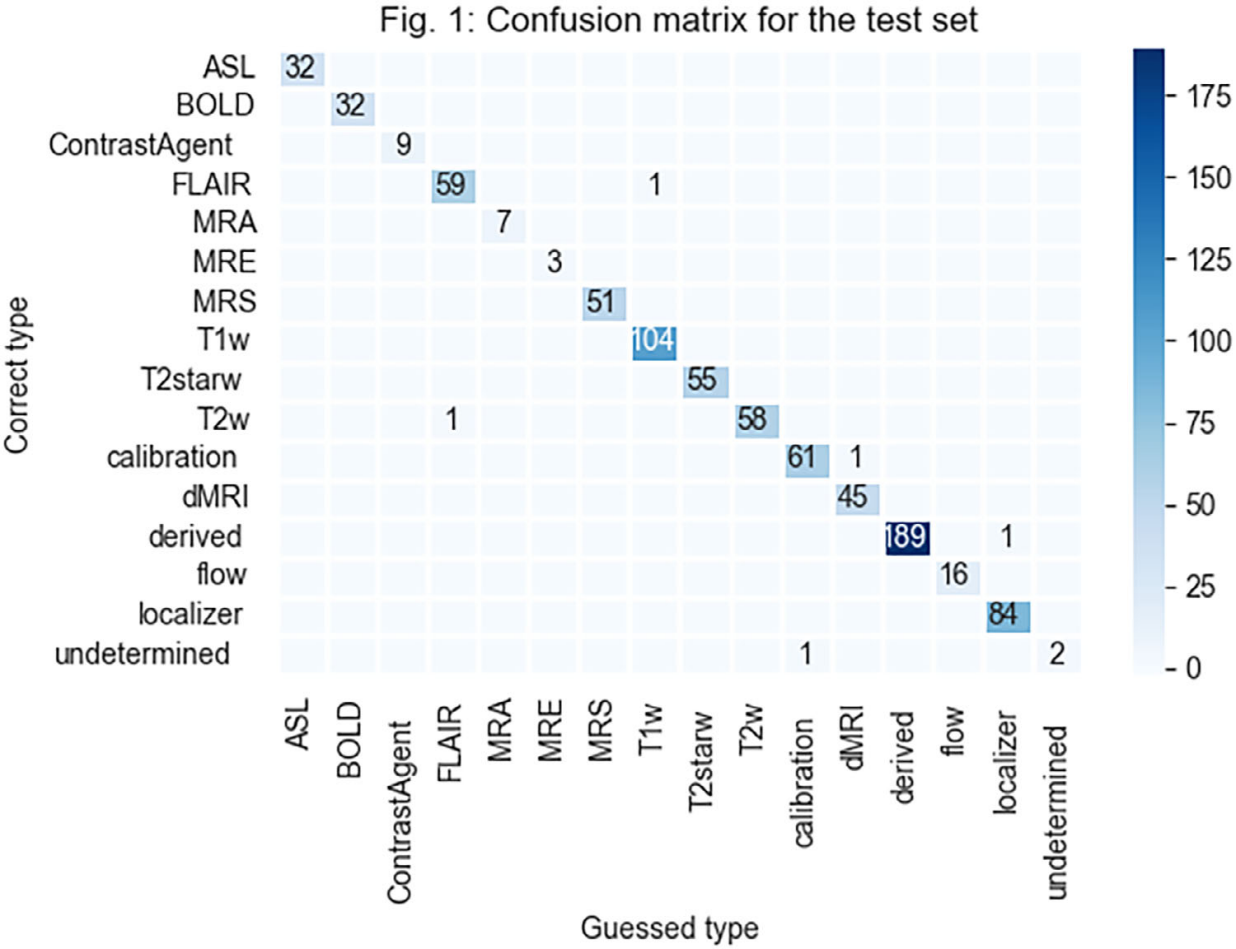# Automatic Classification of Head MR Series

**DOI:** 10.1002/alz70862_109882

**Published:** 2025-12-23

**Authors:** Robert I. Reid, Robel K Gebre, Michael G. Kamykowski, Matthew L. Senjem, Arvin Arani, Petrice M Cogswell, Burcu Zeydan, Orhun H. Kantarci, Kejal Kantarci, Prashanthi Vemuri, Clifford R. Jack

**Affiliations:** ^1^ Mayo Clinic, Rochester, MN USA; ^2^ Department of Radiology, Mayo Clinic, Rochester, MN USA

## Abstract

**Background:**

Neuroimaging data typically arrives as Digital Imaging and Communications in Medicine (DICOM) files, batched by exam. We use *exam* or *study* to denote a single imaging session of a participant, containing up to ∼40 *series* (the generic term for scans and derived products). Different series have different properties and applications, making their labeling complex. Filenames often change in transit, and Series Description tags (0008, 103E) are arbitrary, operator‐entered, and unreliably coupled to the series type. Furthermore, label preferences vary from site to site, making the application of a common classification system mandatory for multisite studies. Here we describe the system that we have developed and applied to the Alzheimer’s Disease Neuroimaging Initiative (ADNI) and SCAN.

**Method:**

Although Series Description is unreliable, DICOM includes other tags that parameterize MR pulse sequences and suggest their purpose. The mapping from these features to types is difficult to explicitly specify, especially since different manufacturers and protocols use different conventions. Thus we developed a machine learning classifier using 4060 semiautomatically labeled head and spinal series from 1.5 to 7T MR scanners, including both site‐specific and standard protocols designed to study a wide range of neurological conditions, including dementia, hydrocephalus, and multiple sclerosis. An 80%/20% split, stratified by type, was used for the training/test sets. After initial training, a few cases in the training set with high errors due to rarity were augmented and the model retrained. Performance was evaluated on the clean test set.

**Result:**

The taxonomy is presented in Table 1. The classifier correctly identified 807 out of 812 (99.4%) series, with the error cases coming from outside the ADNI or SCAN protocols (Table 2). The confusion matrix is shown in Figure 1.

**Conclusion:**

We implemented an automatic series type classifier with labels chosen to be generally useful for the brain MRI community. Some errors were noted, but the test set was deliberately challenging and the errors appear to be ones that could easily be made by a human. The classifier is expected to have good tolerance for as yet unseen head MR protocols but is likely unsatisfactory for body MRI.